#  Evaluating the Frequency of Errors in Preparation and Administration of Intravenous Medications in Orthopedic, General Surgery and Gastroenterology Wards of a Teaching Hospital in Tehran

**Published:** 2013

**Authors:** Mohammad Abbasinazari, Azita Hajhossein Talasaz, Zahra Mousavi, Samaneh Zare-Toranposhti

**Affiliations:** a *Department of Clinical Pharmacy, School of Pharmacy, Shahid Beheshti University of Medical Sciences, Tehran, Iran. *; b *Anesthesiology Research Center, Shahid Beheshti University of Medical Sciences, Tehran, Iran. *; c *Department of Clinical Pharmacy, Faculty of Pharmacy, Tehran University of Medical Science, Tehran, Iran. *; d *Department of Toxicology and Pharmacology, Pharmaceutical Sciences Branch, Islamic Azad University, IAUPS, Tehran, Iran. *

**Keywords:** Administration, Clinical Pharmacist, Intravenous drugs, Medication error, Preparation

## Abstract

The aim of this study was to determine the frequency of medication errors happened during the preparation and administration of intravenous (IV) drugs. This study was designed as prospective cross-sectional evaluations by direct unconcealed observation in a setting consisted of orthopedic, general surgery and gastroenterology wards of a teaching hospital. Participants were those patients hospitalized in these wards along with nurses responsible for preparation and administration of IV medications. Medication errors occurred in the process of preparation and administration of IV drugs, were recorded by a pharmacist. The frequency of medication errors with suggesting a solution to overcome was the main outcome of this study. Details of the preparation and administration stages of the observed drugs were compared to an instructed checklist prepared by an expert clinical pharmacist. From a total of 357 preparation and administration episodes, the most common type of error (%20.6) was the injection of bolus doses and infusion faster than the recommended rate. Metronidazole had the highest rate of error (%24.3). IV rounds conducted at 12 p.m. had the most rate of error (%26.3). Errors happened in the administration process were more prevalent than those in the preparation. No significant correlation was found between the frequency of errors and nurses’ demographic data. This study revealed that the errors happened in the preparation and administration of IV drugs is prevalent. Improving the medication safety by the implementation of clinical pharmacists’ prepared protocols at the point of care is an important concern.

## Introduction

Medication errors are defined as any avoidable happening that may result in improper use of medications or hazards for the patient for which the responsible person may be the health care professional, patient or consumer (1). Medication errors can happen during different stages of the drug delivery process, which have been classified as prescribing, transcribing, dispensing and administrating (2). In USA, it was estimated that more than a million injuries and 44000-98000 deaths annually are related to suboptimal care or errors made by health care professionals (3). These errors are also an important cause of adverse events (4). Each error can result in an estimated $5000 in costs not considering legal expenses (4). Less is known about the medication errors in other countries such as the Middle East (5). Studies on medication errors dated as far back as the 1960s by Barker and McConnell who reported that medication errors occurred much more frequently than that could be obtained via event reports with about 16 errors per 100 doses (6). Bates *et al. *reported that %1 of all adverse drug events were fatal, %12 were life threatening, %30 were serious and %57 were significant (7). In another study, %40 of all events was due to the errors in the stages of administering drugs. Evaluating the influence of automated drug dispensing on the rate of error has shown that errors have not been reduced by advances in technology (8). The number of unintended events including medication errors between various rounds was reported by Capuzzo *et al*. Personal neglect (%86.1), intense workload (%37.5) and new staff (%37.5) were the three important causes of medication errors that nurses were responsible for them in a survey (9). Single site studies in the UK and USA has been shown that nurses make mistakes in preparing and administering IV drugs in %13-84 of all cases (10, 11). Administering a bolus dose too quickly, lack of medications’ checking for expiration date, not controlling the accuracy of prescribed medication, its dosage, the patient who the medication would belong to and not considering hygiene rules, were the major errors observed in practice and mentioned in studies (12-14).

In an overview of IV-related drug administration errors during 5 years from 73769 reported errors, %2.92 to %5.03 of them were associated with harms to patients. Hazardous errors were primarily those containing incorrect concentration and calculations (15). In a research evaluating medication errors in UK, French and German hospitals, the incorrect diluents was utilized in 1%, 18% and 49% of medications used intravenously in each hospital respectively (16). It was shown that there is a direct relationship between the hospital stay and risk of errors. 

Intravenous administration of medications is one of the most common routes in hospitals particularly for those with a long length of hospitalization. Therefore, hospitalized patients are at high risk for adverse drug events (17). Perhaps one of the most serious adverse events that can happen as a result of medication error is one that involves the IV route of administration as Taxis *et al*. estimated that about one half of medication errors occurred in IV preparations and administrations, %1 of which resulted in serious adverse events (18).

Currently, in most Iranian hospitals, the nurses in the wards prepare IV drugs and unfortunately pharmacists are not involved in the medication preparation process. Therefore, there are not any controls for preparations of medications in hospitals. The objectives of present study are to determine the frequency and types of errors, which occur in the preparation and administration of commonly-used IV medications in three different wards of a teaching hospital in Tehran, Iran.

## Experimental

The study was conducted in one of the largest teaching hospitals in Tehran with 620 inpatient beds from July to November 2009. Orthopedic, general surgery and gastroenterology wards were selected for having the highest rate of IV medication prescription as reported by central pharmacy of the hospital during previous 3 months.

Pharmacists at this specific hospital had no role in IV-drug preparation and administration. All IV drugs were prepared and administered in the wards by nursing staff. The nursing staffs were not aware of the purpose of the study or the reason for the presence of a pharmacist as an observer. The study was approved by the local ethics committee and performed in compliance with Helsinki declaration.

Data were gathered over a period of 20 days. Observation days and rounds were alternated *i.e. *randomly selected from all rounds and during all working shifts. IV-drug preparation and administration rounds were done at 6 a.m. (morning), 12 a.m. (noon), 6 p.m. (afternoon) and 12 p.m. (midnight).

Medication errors were detected by direct observation, according to the method established by Barker and McConnell (6). Although different methods have been utilized to evaluate the medication administration errors (MAEs), the observation-based method developed 40 years ago by Barker and McConnellis was commonly accepted as the most reliable.

An educated pharmacist accompanied nurses in the wards during the IV-drug preparation and administration shifts. To maintain the observer reliability, the same observer collected data on randomly selected days during the investigation. If an error was likely to lead imminent harm, the observer would intervene during the observation stage.

Twenty-eight of the most commonly used drugs in the wards were selected for observational evaluation. The selected drugs were determined using pharmacy database. All types of IV drug dosing were involved *i.e*. fixed dosing, PRN (Pro Re Nata; *i.e. *as needed), infusion and intermittent therapy. A checklist for each selected medication was prepared based on the manufacturer leaflet and reference books by an expert clinical pharmacist (19-21). The following items were included in the checklists: correct time for injection, wearing gloves, using proper disinfectants, incorporating the compatible diluting agent, using the correct amount of diluents, considering the compatibility of medications with each other in the same IV line, paying attention to appropriateness of infusion rates and bolus injections, injecting the exact content of medication allocated for the patient as prescribed, controlling the site of injection for probable phlebitis.

After completing the observations, demographic data of nurses including age, sex, work experience and marital status were obtained from the nursing management office of the hospital. The identities of the nurses participated in the study remained confidential. Each nurse was given a code, which was known by the observer only.

## Results

**Figure 1:**
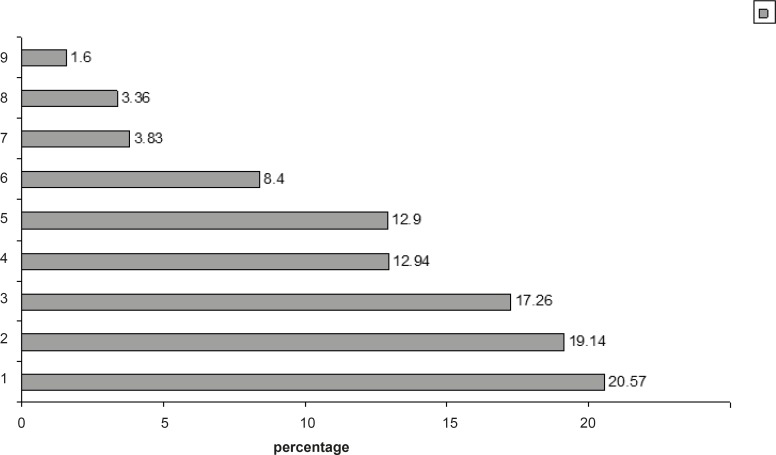
Distribution of IV drug preparation and administration type of errors in the study.

The pharmacist in this study evaluated a total number of 357 preparations and administrations. Each of the 28 most commonly prescribed medications had at least one error during the preparation and/or administration. The majority of errors occurred as wrong infusion and bolus rates (%20.6). Figure 1 depicted the distribution of errors.

1. Wrong infusion/bolus rate.

2. Not to disinfect vials.

3. Not to wear gloves during the preparation and administration.

4. Not to watch patients for possible phlebitis.

5. Using inappropriate diluents for solving.

6. Using incorrect dose of diluents.

7. Incorrect time for injection.

8. Not to inject the content completely.

9. Incompatibility between the medications.

In the evaluated medications, Metronidazole was the most common drug involved in errors (%24.3), followed by Ranitidine (21.8%), Ceftriaxone (18.9%), Cefazolin (18.1%) and Imipenem cilastatin (16.9%). Considering the working shifts, most percentage of errors occurred at midnight (12 p.m) (%26.3), followed by morning (6 am) (26.2%), noon (12 a.m) (23.9%), and finally afternoon (6 p.m.) (23.6%).

In Table 1, the number of nurses, beds and ratio of nurses to bed in observed wards is shown. The frequency of medication errors at different wards were as follows: general surgery (%35.6), gastroenterology (32.5%) and orthopedic (31.9%).

**Table 1:** Number of nurses, beds and nurse/bed ratio in studied wards. *: The standard for this ratio is 1:5 in medical wards

**Ward**	**Number of nurses**	**Number of beds**	**Number of Nurse/bed Ratio** ^*^
**Orthopedic**	12	49	0.24
**General surgery**	9	43	0.20
**Gastroenterology**	9	42	0.21
**Total**	30	134	0.22

Thirty nurses responsible for the administration and preparation of medications were observed during the study period. The included nurses consisted of 9 males (%30) and 21 females (%70), with a mean age of 34.6 years old, mean work experience of 10.5 years, and 19 (%63.4) of them were married.

No significant correlation was found between the frequency of errors and nurses demographic data (in each case p > 0.05).

## Discussion

The results of the present study showed a relatively high rate of error in the preparation and administration of commonly used IV medications in an Iranian teaching hospital. The high percentage of identified errors must be viewed in light of the detailed and systematic examination of errors and types of error at each stage of the medication process. Errors including wrong dose, expired drugs usage and administrating the medication to the wright patient were not evaluated in our study. The observer only assessed the steps in preparation and administration of the common IV medications administered in three wards. This may be one of the reasons for inconsistency between our findings with other studies. Reported drug administration errors varied in the wide range of %0.6 to %27 in previous studies (22, 23). This different rate of observed errors can be explained partially by applying different definitions and settings. A similar previous study indicated harm rates resulting from error to be at the range of %2.92-%5.03 (15). Patients themselves intercepted some of the errors and hence patient’s education is also important to prevent medication errors. In our study, none of the identified errors resulted in adverse effects or major risk for the patient.

The majority of errors in our study occurred in the shift of 12 p.m. Higher nursing workloads, sleepiness and tiredness may distract them and influence their ability to concentrate, which in turn results in an increase in chance of errors (8). One possible solution is to increase the number of ward staff. In our study, the most errors were observed in general surgery, gastroenterology and orthopedic, respectively. As shown in Table 1, the ratio of nurses to beds in these wards declined concordantly. This could justify the reason behind the prevalence of errors in general surgery compared to the other wards. The results of our study are also consistent with that of Taxis *et al. *who found the most common type of errors as injecting bolus doses and infusion more rapidly than the recommended rate (18). The recommended bolus administration time according to the references applied in our study was between 3 and 5 min. Administration of drugs faster than the recommended was the most prevalent error. Inappropriate storage of diluted drug and incompatibilities were minor errors occurred in our setting. The low rate of these medication errors can be explained by the fact that in this hospital, the pharmacy personnel routinely inspect all approved medication storage areas. Furthermore, nurses are careful to avoid mixing multiple medications in a syringe or solution or to deliver drugs from separate IV line if possible. Although proper methods of preparation and administration of parenteral drugs are important to prevent thrombus formation, hypersensitivity reactions and infections (24), nurses involved in our study forget to monitor the site of injection for probable reactions.

For any type of medication error, the observer can have some form of intervention. Nevertheless, we tried to avoid as much interventions as we could to prevent observer-induced bias. Although the nurses were unaware of the reasons for observation, they were aware that they were being observed. This may have biased our findings, and therefore, our estimates of error may be conservative.

Our study was not without limitations. First of all, our study lacked validation of the tool used to determine error. Our tool was developed using the manufacturers’ guidelines and three reputable references. Observation per se may also affect practice and result in a decrease of medication error rate.

Secondly, it has been shown that data collection periods of more than nine consecutive days may necessitate the involvement of more observers (25). To overcome this obstacle in some part, the observer in our study collected data based on randomly selected days.

As a future perspective, since medication preparations and administrations are the last line of defense against medication errors, identifying and implementing a plan for improvements in this aspect is the next step in the process. One obvious solution to aid in the process would be to consider pharmacy involvement in product preparation by implementing protocols prepared by clinical pharmacists or establishment of reporting error systems. Nevertheless, more research into optimizing the drug preparation and delivery systems should be conducted to minimize the chance of error and harm to patients. Further research is required on issues such as nursing administration policy and potential contributing factors.

In conclusion, this study indicates the frequency of drug administration errors in developing countries such as Iran. Higher rates than recommended in bolus and infusion were the most common type of drug administration errors. In a study, Abbasinazari *et al*. have shown that clinical pharmacist can play a significant role in nurse training as an effective method to reduce drug-food interactions in hospitals (26). Involvement of a pharmacist can be a solution to reduce the rate of errors by training the health care professionals and establishing a non-punitive system of reporting medication errors to encourage documenting the information and implementing the risk management protocol.
